# Peroxiredoxins—The Underrated Actors during Virus-Induced Oxidative Stress

**DOI:** 10.3390/antiox10060977

**Published:** 2021-06-18

**Authors:** Inna L. Karpenko, Vladimir T. Valuev-Elliston, Olga N. Ivanova, Olga A. Smirnova, Alexander V. Ivanov

**Affiliations:** Center for Precision Genome Editing and Genetic Technologies for Biomedicine, Engelhardt Institute of Molecular Biology, Russian Academy of Science, 119991 Moscow, Russia; ilkzkil@gmail.com (I.L.K.); gansfaust@mail.ru (V.T.V.-E.); olgaum@yandex.ru (O.N.I.); o.smirnova.imb@gmail.com (O.A.S.)

**Keywords:** peroxiredoxin, reactive oxygen species, hydrogen peroxide, chaperone, virus, biomarker

## Abstract

Enhanced production of reactive oxygen species (ROS) triggered by various stimuli, including viral infections, has attributed much attention in the past years. It has been shown that different viruses that cause acute or chronic diseases induce oxidative stress in infected cells and dysregulate antioxidant its antioxidant capacity. However, most studies focused on catalase and superoxide dismutases, whereas a family of peroxiredoxins (Prdx), the most effective peroxide scavengers, were given little or no attention. In the current review, we demonstrate that peroxiredoxins scavenge hydrogen and organic peroxides at their physiological concentrations at various cell compartments, unlike many other antioxidant enzymes, and discuss their recycling. We also provide data on the regulation of their expression by various transcription factors, as they can be compared with the imprint of viruses on transcriptional machinery. Next, we discuss the involvement of peroxiredoxins in transferring signals from ROS on specific proteins by promoting the oxidation of target cysteine groups, as well as briefly demonstrate evidence of nonenzymatic, chaperone, functions of Prdx. Finally, we give an account of the current state of research of peroxiredoxins for various viruses. These data clearly show that Prdx have not been given proper attention despite all the achievements in general redox biology.

## 1. Introduction

Viral infections are given more and more attention in recent years, especially during the pandemic of severe acute respiratory syndrome-associated coronavirus 2 (SARS-CoV-2) that was first reported in China at the end of 2019. [1]. This pandemic clearly showed viruses can impose a significant threat to humans and become a tremendous burden on healthcare systems and the economy of all countries. In recent decades there has been a tremendous success in the investigation of the biology of several widely spread viruses and in the development of highly effective antiviral agents. One example is the hepatitis C virus, whose life cycle has been thoroughly investigated. Several antiviral agents that can cure almost all chronic hepatitis C virus patients have been discovered and introduced into clinical practice [2,3,4]. Similar progress is being achieved for hepatitis B virus infection, for which a series of very promising molecules are at the 2D and 3D stage of clinical trials [5]. Of course, rapid progress can be seen in many areas of investigation of SARS-CoV-2, such as the development of infectious models, design of effective vaccines, and investigation of mechanisms by which the infection can trigger cytokine storms. However, there is still much unknown about the mechanisms of pathogenicity of many viral infections, which merits further studies.

Enhanced production of reactive oxygen species (ROS) and dysbalanced antioxidant response that are often referred to as oxidative stress were shown to be the hallmarks of many viruses from various families. They include hepatitis B and C viruses, human immunodeficiency virus (HIV), various respiratory viruses [6,7,8,9], herpesviruses, etc. [10]. There are multiple lines of evidence that redox disbalance in the infected cells contributes to the development of various virus-associated pathologies, including inflammation, fibrosis, metabolic alterations, and even cancer. Such conclusions are drawn from established correlations between markers of oxidative stress and the degree of a respective pathology or rate of its occurrence. Noteworthy that these pathologies may include not only the ones that are developed in the infected tissue/organ: For example, HIV-associated dementia is considered to be triggered by oxidative stress in brain tissues induced by the virus Tat protein [11]. Oxidative stress can also be a direct factor of carcinogenesis, as shown in mice overexpressing HCV core protein that developed liver tumors in the absence of inflammation, the most known precancerous event [12].

Despite rapid advances in general redox biology, many of its achievements and concepts are being neglected by virologists. First, most studies in the field focus on general levels of ROS production and antioxidants without paying proper attention to their localization. The most pronounced example is catalase, the most studied antioxidant enzyme by infectionists: This enzyme is localized in the peroxisome, and this factor (together with low affinity to its substrate) does not allow it to play a major role in scavenging hydrogen peroxide outside this organelle. The second is a concept of redox switches—i.e., the proteins whose cysteine residues are selectively modified by peroxide with a change in protein’s functions [13]. In the case of Src, PP2a, and STAT3, it was shown that enhanced ROS production may selectively affect signaling pathways, pointing to H_2_O_2_ as a signaling molecule [14,15].

Our review focuses on a family of peroxiredoxins (Prdx) that can act both as potent ROS scavengers in various organelles and as signaling proteins. It consists of six members, but neither of them has been regarded as an antioxidant during virus-induced oxidative stress. Thus, our goal was to present general knowledge about these proteins for virologists and to summarize current data on their status and functions during viral infections to promote further research in this area.

## 2. A Family of Peroxiredoxins

Peroxiredoxins (Prdx’s) are the enzymes that efficiently scavenge hydrogen peroxide and organic peroxides, thus protecting cells against ROS accumulation and playing a role in redox-dependent signaling pathways. In mammalian cells, this family of enzymes is comprised of six isoforms. Prdx1 is expressed mainly in the cytoplasm [16] and in the nucleus (Table 1) [17]. Prdx2 is found not only in cytoplasm [16,17], but also in nucleus [16,18]. Under oxidative stress, it can also be shown to bind the plasma membrane, at least in erythrocytes [19]. Prdx3 is addressed to mitochondria [16]. Peroxiredoxin 4 exists primarily as an ER-residing protein [20,21], sometimes also found in the lysosomes [16], mitochondria [16], and Golgi apparatus [21]. This enzyme can also be excreted from the cells [21]. Prdx5 is localized to several compartments: Mitochondria [16], peroxisomes, and cytoplasm [22], and such variability is attributed to the existence of several isoforms resulting from alternative mRNA splicing [23]. Finally, Prdx6 is mainly expressed in acidic organelles [24]; in some cases is also found in mitochondria and nuclei [16]. Changes in its localization can occur in response to some stimuli, such as ischemia/reperfusion [25].

Peroxiredoxins are widely expressed in various tissues, though in different types of cells. The highest levels of Prdx1 expression were found in kidneys, liver, and lung, whereas it is also present in other tissues [26]. Prdx2, Prdx3, and Prdx5 have ubiquitously expressed enzymes [22,26], whereas Prdx4 is more highly expressed in the pancreas, spleen, liver, testis, and lungs compared to other organs [21]. The highest levels of peroxiredoxin 6 were found in the lung, whereas it was also detected in the brain, heart, liver, spleen, kidney, and testis [27]. A systematic investigation of the expression of all six Prdx isoforms in various mouse organs was presented in an outstanding work of J.R. Godoy et al. [28]. Specifically, they paid special attention to disseminating the expression between various types of cells within a tissue. It was shown that almost every organ expresses every peroxiredoxin, though at a different level and in different types of cells.

## 3. Mechanism of Oxidation and Reduction of Peroxiredoxins

All peroxiredoxins share a conservative peroxidate cysteine residue (Cys_p_, N-terminal Cys) located in the N-terminal part of the protein that is crucial for catalysis. It is selectively oxidized by peroxide into cysteine sulfenic acid (Cys–SOH). However, the subsequent reactions for this residue are different for various peroxiredoxins. Depending on them, the members of this family are divided into three subfamilies [29,30]. Peroxiredoxins 1–4 comprises a group of typical 2-Cys enzymes. They have an additional cysteine referred to as a resolving (Cys_r_). It interacts with the cysteine sulfenic acid of another, oxidized, Prdx molecule leading to the formation of a homodimer with an intermolecular disulfide bond. In the case of Prdx5, an atypical 2-Cys enzyme, the resolving cysteine interacts with an oxidized cysteine within the same polyprotein yielding an intramolecular S–S bind. The reduction of oxidized peroxiredoxins is achieved by either cytoplasmic thioredoxin 1, or mitochondrial thioredoxin 2, depending on their localization. Prdx6, the only mammalian member of the 1-Cys subfamily, does not possess Cys_r_ residue. Its oxidized peroxidate cysteine reacts with glutathione (GSH)-saturated glutathione-S-transferase π (GSTπ), forming a Prdx6:GST π (GSH) heterodimer [31,32]. Noteworthy that this dimerization is mediated mostly by hydrophobic interactions rather than from the formation of a mixed disulfide [31]. This leads to S-glutathionylation of the peroxiredoxin 6, an essential step in its reactivation [33]. Hence, the peroxidase activity of this enzyme is referred to as glutathione peroxidase activity. Prdx6 exhibits two enzymatic activities: Peroxidase and phospholipase, and the heterodimerization and S-glutathionylation are required for peroxidase activity only [31,34]. 

A cysteine sulfenic acid is not a terminal product of oxidation of peroxiredoxin by peroxides. In their excess, the peroxidate Cys–SOH can be further oxidized into sulfinic acid (Cys–SO_2_H). Such hyperoxidation was shown for both 2-Cys and 1-Cys peroxiredoxins [35]. In this state, peroxiredoxins become inactive. Mitochondrial Prdx3 is more resistant to hyperoxidation than cytoplasmic Prdx1 and 2, which may prevent it from inactivation in a more oxidized environment [36,37]. Sulfinic acid cannot be reduced by the thioredoxin system. In contrast, hyperoxidized 2-Cys peroxiredoxins are converted back into sulfenic form by another enzyme-sulfiredoxin [38,39,40]. The speed of this regeneration is different for various peroxiredoxins: The fastest regeneration occurs for Prdx2, whereas the slowest—for Prdx3 and 6 [41]. Moreover, Kim et al. suggested that hyperoxidation of Prdx6 is irreversible [35]. Finally, sulfinic groups of peroxiredoxins can be reduced by sestrins, as shown at least for bacterial enzymes [42], albeit their role as reductases for mammalian Prdx remains doubtful [43].

The first Prdx1, 2, and 3 have been shown to possess peroxidase activity and to rely on thioredoxin (Trx) as a source of reducing equivalents. None of the three proteins exhibited peroxidase activity in the presence of glutaredoxin. All three enzymes showed similar kinetic properties: The Vmax was 6–13 μmol/min per mg at 37 °C, the K_M_ for Trx was 3–6 μM, and the K_M_ for H_2_O_2_ was <20 μM [44].

It has been demonstrated that Prdx3 is not the only substrate for thioredoxin 2 (Trx2), but can also be reduced by glutaredoxin 2 (Grx2) via the dithiol reaction mechanism. Grx2 reduces Prdx3 exhibiting catalytic constants (K_M_ 23.8 μM; Vmax, 1.2 μmol/mg/min similar toTrx2 (K_M_ 11.2 μM; Vmax, 1.1 μmol/mg/min. The reduction of the catalytic disulfide of the atypical 2-Cys Prdx5 is limited to the Trx system.

The results presented here provide strong evidence that both mitochondrial redox systems, Grx2 and Trx2, contribute to the redox state of the typical 2-Cys Prdx3 in vivo, but not to the reduction of the atypical 2-Cys Prdx5, which appears to rely on the Trx system for the reduction of the disulfide formed during its reaction cycle [45].

## 4. Peroxiredoxins Are Highly Effective Scavengers of Peroxides and Peroxinitrite

Peroxiredoxins are highly efficient scavengers of peroxides. Outside the redox biology community, neutralization of hydrogen peroxide is attributed mostly to low molecular weight compounds termed as antioxidants (glutathione, etc.). However, their efficacy is very low. For example, the bimolecular rate constant (k_+1_) for the reaction of glutathione thiolate anion and H_2_O_2_ is ~10^1^ M^−1^·s^−1^ [46]. In contrast, such constants for catalase reach 10^7^ M^−1^·s^−1^, whereas for glutathione peroxidases—up to 10^8^ M^−1^·s^−1^ (as reviewed for mitochondrial peroxidases by the authors of [47]). In the case of peroxiredoxins, the rate constants for a reaction between reduced Prdx and ROOH lie between 2 × 10^3^ and 4 × 10^7^ M^−1^·s^−1^, depending on the nature of peroxide and peroxiredoxin isoform [48]. Peroxiredoxins 2, 3, 4, and 6 exhibit comparable efficacy of scavenging hydrogen peroxide: The reaction constants being app 10^7^–10^8^ M^−1^·s^−1^ [49,50,51,52]. Prdx5 is less effective in neutralizing H_2_O_2_ (k_2_ = 3 × 10^5^ M^−1^·s^−1^), instead it has more pronounced activity towards organic alkyl peroxides (k_2_ = 5 × 10^6^ M^−1^·s^−1^) [53]. The large in-put of peroxiredoxins into peroxide neutralization is also based on their high levels in intracellular compartments. For example, the Prdx3 level in mitochondria reaches 60 μM, which is 30-fold higher than that of GPx1 or GPx4 ([44,54] and reviewed by the authors of [47]). Thus, Prdx3 accounts for the elimination of 90% hydrogen peroxide in this organelle. Similarly, a dramatic difference between efficiencies in peroxide neutralization between Prdx4 and GPx7/8 underlies a predominant role of the former in protection against peroxides in the endoplasmic reticulum (ER) [50,55]. The level of Prdx2 in the cytoplasm of erythrocytes reaches as much as 250 μM, giving its ability to scavenge an equimolar amount of H_2_O_2_ even without the need for recycling [56]. Finally, another critical property of peroxiredoxins is their ability to act at low levels of peroxides. For example, K_M_ for hydrogen peroxide in the case of catalase is 80 mM [57], and for bovine erythrocyte GPx is 240 μM [58]. In contrast, K_M_ values for Prdx6 is 25 μM [59], whereas for Prdx1-3 they are below 20 μM [44]. Actually, there are even lower than these values. For example, Low et al. observed that Prdx2 effectively neutralizes H_2_O_2_ even at submicromolar concentrations [56].

Peroxiredoxins scavenge not only hydrogen, organic, or lipid peroxides. They can also neutralize peroxinitrite, a product of a reaction between nitric oxide and superoxide anion. Initially it was shown for a bacterial peroxiredoxin [60], and later expanded to mammalian Prdx2 [51], Prdx5 [61], and Prdx6 [52]. In the case of Prdx5, the second rate constant for peroxynitrite neutralization is 7 × 10^7^ M^−1^·s^−1^ which is two orders of magnitude higher than the constant for hydrogen peroxide (see above) [53,61]. Thus, peroxiredoxin 5 is often referred to as a peroxinitrite reductase. Prdx2 has a similar efficiency for peroxinitrite scavenging [51]. The catalytic cycle of peroxiredoxins during HNOOH neutralization is similar to that for peroxides. Moreover, an excess of peroxinitrite can also cause hyperoxidation of the enzyme as well as nitration, with both these modifications causing Prdx inactivation [51].

## 5. Transcriptional Regulation of Peroxiredoxin Expression

To understand the possible interference of viral infections in the status of peroxiredoxins in infected cells, principles of regulation of Prdx expression should be presented. The expression of peroxiredoxins is not unregulated. To date, a wide array of transcription factors have been shown to regulate transcription of their genes (Table 2). They include Nrf2 [62], subunits of NF-κB [63], cMyc [64], AP-1 [65], specificity protein 1 (Sp1) [66], CCAAT/enhancer-binding protein β (C/EBPβ) [67], FoxO3a [68], glucocorticoid receptor [69], PGC-1α [70], GATA-binding protein 1 [71], nuclear respiratory factors 1 [72] and 2/GABP [73], as well as repressors, such as KLF9 [74,75] or Nrf3 [76]. 

The Nrf2 factor has attributed most attention as a regulator of Prdx expression. To date it has been shown to control transcription of peroxiredoxins 1 [77], 3 [78], 5 and 6 [76], although for Prdx2 and Prdx5 [69] contrary data also exist. Prdx1 promoter contains two electrophile response elements (EpRE), thus binding Nrf2 after hypoxia/oxygenation [77]. Indeed, its expression strongly correlates with the expression and activation of Nrf2 in vivo [62]. The Prdx3 factor is another gene that is clearly induced by the Nrf2 [78,79]. However, for the other isoforms of these antioxidant enzymes, the data are less straightforward. For example, the Prdx5 promoter contains several potential binding sites for activator protein 1 (AP1), NF-κB, Nrf2 (antioxidant response element, ARE), as well as Glucocorticoid (GRE) and Insulin (InRE) Response Elements [23]. At the same time, Kropotov et al. found that neither basal expression of Prdx5 is regulated by Nrf2, nor in response to the stress triggered by menadione [69]. Prdx6 was also considered a classical Nrf2-dependent gene, since its promoter contains ARE sequence, and gene transcription can be upregulated in response to H_2_O_2_ treatment [76]. A ROS-independent induction of Prdx6 via Nrf2 was also reported for cells treated with keratinocyte growth factor that triggers Nrf2 translocation to the nucleus [80]. However, Nrf2 can also suppress the expression of this enzyme via ARE-dependent induction of the KLF9 protein that binds to the RKBE sequences in the Prdx6 promoter, leading to repression of the latter [74,75]. Noteworthy that the Nrf2 factor also controls the expression of sulfiredoxin [81,82]. Thus, activation of the Nrf2/ARE pathway (for example, in response to hyperoxia) leads to significant induction of Srx, preventing Prdx hyperoxidation, and therefore, protecting cells during enhanced ROS production.

Expression of Prdx6 is controlled by Sp1 and C/EBPβ transcription factors [66,67] that are implicated in the control of cell differentiation and proliferation, and as a result, in tumorigenesis [83,84]. The involvement of Sp1 was demonstrated in cells treated with natural compound curcumin that affects redox pathways [66]. As for the C/EBPβ, it acts in a complex with the cAMP response element-binding protein (CREB) [67]. Finally, Prdx6 gene transcription is affected by dexamethasone, pointing to glucocorticoid receptor as another regulatory factor [69].

Another transcription factor that controls the expression of several peroxiredoxins is FoxO3a that has multiple functions in a cell and whose functions are modulated by various protein kinases that affect its intracellular localization and activity [85]. To date it is accepted that FoxO3a regulates expression of Prdx 2 [86], 3 [68,70,87], and 5 [70]. In the case of peroxiredoxin 3, this transcription factor controls both constitutive and inducible expression. Induction of Prdx3 in response to cytotoxic agents, such as doxorubicin, is achieved via downregulation of FoxO3a phosphorylation [68] which is typical for its control by the PI3K/AKT pathway [85]. In addition, expression of Prdx3, as well as Prdx5, is regulated by FoxO3a in a complex with the mitochondrial fasting-inducible peroxisome proliferator-activated receptor gamma coactivator 1-alpha (PGC-1α) and in its turn is controlled by the sirtuin 1 (SirT1) [70].

The roles of other transcription factors in controlling peroxiredoxin expression are less clear. It has been reported that Prdx3 and 5 can be induced during quercetin-triggered oxidative stress by the nuclear respiratory factor 1 (Nrf1) [72]. Nuclear respiratory factor 2/GABP also controls basal expression of Prdx5 [73]. This isoform of peroxiredoxins is also controlled by GATA-binding protein 1 (GATA1) [71], the transcription factor that is involved in cell differentiation and is strongly associated with the development of various tumors [88]. Prdx1 was also shown to be induced via the activating protein 1 (AP-1) [65] and RelA/p65 subunit of the NF-κB in a nonclassical fashion [89], while Prdx3—via cMyc [64]. The classical NF-κB pathway has been shown to control the expression of Prdx2 [63].

## 6. Peroxiredoxins as Regulatory Proteins

The role of peroxiredoxins in a cell is not restricted to the scavenging of peroxides, thus counteracting their enhanced production. They can exhibit regulatory functions by two mechanisms: Through specific oxidation of cysteine groups of other members of cell signaling pathways or by acting as molecular chaperones.

### 6.1. Peroxiredoxins as Sensors on Hydrogen Peroxide That Trigger Specific Oxidation of Cell Proteins

2-Cys peroxiredoxins can form intermolecular disulfide bonds with a wide array of other cellular proteins, including transcription factors, protein kinases, and phosphatases, chaperones, metabolic enzymes/transporters, actors of the translational machinery, and many others [90]. This is due to the unique ability of peroxiredoxin to sense hydrogen peroxide, making them the main sensor of ROS in a cell [91].

A critical parameter in transferring oxidative equivalent to another protein is the stability of the sulfenic group of a peroxiredoxin. It has been shown that a lower rate of resolution step of a catalytic cycle for Prdx2 compared to Prdx1, leading to higher stability of its -SOH group, forms intermolecular disulfides with other proteins for their subsequent oxidation [92]. One of the most known examples is the interaction of Prdx2 with the STAT3 transcription factor [93]. Here Prdx2 is responsible for sensing H_2_O_2_ and passing oxidizing equivalents to the transcription factor, leading to the formation of disulfide bonds between monomers to form an oligomer with reduced transcriptional activity. This interaction is specific, as it occurs on membranes prior to oxidation of peroxiredoxin, and is further stabilized by the Annexin A2 protein [94]. Formation of this complex ensures rapid sensing of peroxides for its immediate channeling to the transcription factor. A similar example was described for yeast Tsa peroxiredoxin that passes oxidative equivalents to the Yap1 transcription factor [95]. Peroxiredoxins also affect the mitogen-activated protein kinase (MAPK) pathway, as both Drosophila enzyme Jafrac1 and human Prdx2 interact with and trigger oxidation of MEKK1 (in the case of a fruit fly) or its human homolog MEKK4 resulting in activation of p38 kinase and the respective signaling [96]. In addition, the abovementioned yeast peroxiredoxin Tsa also triggers an oxidation-dependent activation of the cAMP-dependent protein kinase (PKA) [97]. Peroxiredoxins can also interact with the cardiac DJ-1 protein, also leading to the formation of intermolecular S–S– bonds [98]. It can be speculated that this oxidation could be required for the interaction of DJ-1 with the apoptosis signal-activating kinase 1 (ASK1), thus making this kinase pathway also redox-sensitive [99]. A similar mechanism was described by Prdx1 and its ability to induce oxidation of a transmembrane protein GDE2, making this peroxiredoxin a regulator of neuronal differentiation [100].

A vast majority of data on the subject concerns the ER-resident peroxiredoxin 4 and its interaction with various protein disulfide isomerases (PDI)—the main players in oxidative protein folding. The ERp44 is one of the main such PDIs that binds to oxidized Prdx4 through thiol-disulfide exchange [101]. Other partners are the Erp46 and P5 isomerases, all of which then use the oxidative equivalents from Prdx4 for forming disulfide bonds in their substrate proteins [102]. These equivalents are likely to be taken from ER-residing oxidoreductase 1α found in a complex with Prdx4 and Erp44 [103]. At the same time, there is data suggesting that binding to Erp46 requires prior hyperoxidation of Prdx4 [104]. One of the most known proteins whose oxidative folding depends on peroxiredoxin 4 is proinsulin in beta-cells [105].

### 6.2. Peroxiredoxins as Chaperones

Peroxiredoxins exist not only as monomers/dimers, but also as high-molecular weight aggregates [106,107,108,109]. These aggregates are presented by decamers or dodecamers formed by dimers via hydrophobic interactions or disulfide bonds, though these bonds are not essential for stabilizing the aggregates [110]. Formation of high molecular weight structures has been shown for Prdx1 [111], Prdx2 [112], Prdx3 [106], Prdx4 [20,113], and Prdx6 [114]. Aggregation is promoted by heat shock [115] and various posttranslational modifications, including peroxidation [113,116], phosphorylation of tyrosine residues [117], lysine acetylation [118], and nitrosylation [119]. However, it should be mentioned that, in some cases, additional oxidation may disrupt decamers into dimers [120]. The additional influence on the stability of these multimers is caused by other factors, such as signaling molecules like phosphatidylserine [121]. In contrast, some other modifications, such as S-glutathionylation trigger the disassembly of decamers into dimers [122].

Formation of (do)decamers is accompanied by a decrease or a loss of peroxidase activity, but gaining of an activity of a chaperone [112,119]. In such a state, they can nevertheless support cell resistance to oxidative stress by promoting the expression of other antioxidant enzymes, such as superoxide dismutase 1 (SOD1) or glutathione peroxidase 1 [111]. In chaperone states, peroxiredoxins also play signaling functions, as can be exemplified by interaction with TLR4 with concomitant production of proinflammatory cytokines [123], or by interaction with ubiquitin C-terminal hydrolase-L1 (UCH-L1), preventing this protein from thermal/oxidant-dependent inactivation [124]. Another example is the interaction of Prdx1 with a DNA damage-responsive factor APE1 that can further interact with the NF-κB, leading to the expression of interleukin 8 (IL-8) [125]. Finally, in a chaperone state, some peroxiredoxins may be exported from a cell—making them biomarkers of oxidative stress [126].

## 7. Peroxiredoxins and Viral Infections

### 7.1. Involvement of Peroxiredoxins in Replication of Viruses

Since peroxiredoxins are multifunctional proteins, it is not surprising that they are critical for the replication of certain viruses. It can be assumed that they regulate life cycle of pathogens not only through modulation of redox homeostasis, but also via interaction with viral protein and playing a role as chaperones [127], although this property in the context of viral infection remains the least explored.

Some viruses suppress or even degrade peroxiredoxins to evade their positive regulation of antiviral pathways. As such, porcine picornavirus and foot-and-mouth disease virus suppress Prdx6, since its PLA activity can inhibit their replication, although by a yet undefined mechanism (Table 3) [128]. Decreased expression of Prdx4 in HIV-infected T-cells can present a way to evade H_2_O_2_-mediated activation of NF-κB that inhibits initiation of the virus long terminal repeat (LTR) replication [129]. Thus, Prdx4 downregulation facilitates ROS-dependent activation of this transcription factor that plays an important role in the transcription of the HIV genome [130,131,132,133].

Prdx1 acts as an antiviral protein towards the hepatitis B virus—presumably by interaction with the HBx protein, as well as with viral RNA [134]. It has been shown that binding of Prdx1 in a complex with exosome component 5 (Exosc5) to the HBV RNA leads to degradation of the latter. It is worth mentioning that since the Prdx1-Exosc5 complex also exists in HBV uninfected cells, this peroxiredoxin could also play antiviral activity in the case of other infections.

Human papilloma and influenza viruses modulate peroxiredoxins to suppress oxidative stress in infected cells. In particular, the E7 protein of human papilloma virus 16 (HPV 16) triggers enhanced expression of Prdx2 [135]. It could be speculated that the effect could be due to the interaction of E7 with TAF-110 protein of the transcription machinery complex for specific promoter modulation [136]. In the case of the influenza virus, silencing of Prdx1 was shown to inhibit virus propagation [127,137]. The ribonucleoprotein of this virus colocalizes with Prdx1 at the plasma membrane of the infected cells, therefore probably preventing oxidative damage of viral RNA or proteins of the virus via its chaperone activity [127]. Definitely, its role merits further studies.

Prdx1 is also a critical component of measles virus replication, as it interacts with the Mev-N protein, a component of RNA replicase [138]. Moreover, Prdx1 competes with the virus Mev-P protein for Mev-N that is synthesized during the late stages of the virus life cycle. Therefore, Prdx1 is likely to be essential for the early stages of infection, such as replication and transcription of the virus genome. Prdx3 was reported to be a critical cellular protein for replication of Junin mammarenovirus, an etiological agent for the Argentine hemorrhagic fever [139].

For respiratory syncytial virus (RSV), peroxiredoxins are required to protect the infected cells from virus-induced oxidative stress and concomitant cell death [9]. As this virus infection leads to the downregulation of several important antioxidant enzymes, Prdx 1 and 4 prevent cellular nuclear cytoskeletal proteins, including annexin A2 and desmoplanin, from oxidation [140]. 

**Table 3:** Effect of peroxiredoxins on replication of viruses.

Virus	Peroxiredoxin	Action	Function	Model	Ref.
HIV	Prdx4	Antiviral	Promotes genome transcription	T-cells from patients	[129]
Porcine picornavirus, foot and mouth disease virus	Prdx6	Antiviral	Unknown	Various cell lines	[128]
Hepatitis B virus	Prdx1	Antiviral	Facilitates degradation of viral RNA	Hepatocarcinoma cell lines	[134]
Influenza virus	Prdx1	Proviral	Prevents oxidative damage of viral RNA/proteins	Primary and immortalized/tumor cell lines	[127,137]
Measles virus	Prdx1	Proviral	Component of replicase	HEK293 cells, low MOI	[138]
Respiratory syncytial virus	Prdx1,4	Proviral	Prevent ROS-induced cell death, protect nuclear cytoskeletal proteins	A549 cells, high MOI	[140]
Junine mammarenovirus	Prdx3	Proviral	Unknown	HEK293, low MOI	[139]

MOI: multiplicity of infection.

To sum up, various viruses affect the expression of peroxiredoxins, although the data are nonsystematic and sometimes contradictory.

### 7.2. Changes in Peroxiredoxin Expression during Virus Infections

Several viruses were shown to affect the expression of peroxiredoxins, although these data are rather nonsystematic. The uncontradictory data are presented in Table 4. Pronouncedly increased expression of Prdx1 and Prdx2 occurs in cells overexpression oncoproteins of the high-risk HPV [135,141]. Similar increased content of Prdx1 was noted in the case of overexpression of Epstein–Barr virus (EBV) EBNA1 protein, albeit no changes in its expression occur in such cells [142].

There is much discrepancy in data on the imprint of the influenza virus on peroxiredoxins, possibly due to different virus isolates and cell lines used by various groups. In the A/WSN/33 and (HPAI) H5N1 strains, the total amount of Prdx1 remains constant, whereas its levels at the membranes of A/WSN/33-infected cells increased [127], and in (HPAI) H5N1-infected-decreased [137]. In mice infected with the H1N1 in A/PR/8/34 [PR8] strain, an exhaustion of Prdx6 levels in lungs was reported, whereas in their alveolas-increased [148]. The same strain also triggers the secretion of Prdx1 and Prdx2 from the infected cells [149]. An increase in Prdx6 expression was also shown for the classical swine fever virus and its NS5A protein in particular [150,151], although opposite data also exist [152]. Another respiratory infection, respiratory syncytial virus (RSV), enhances the expression of Prdx2 [153]. Data on the effect of coronaviruses on peroxiredoxins remain vague, although expression of Prdx3 during SARS-CoV-2 expression could be affected [154].

For flaviviruses, the situation is also not quite clear. Peripheral blood mononuclear cells (PBMC)s from patients with dengue virus exhibit enhanced expression of Prdx1 [143]. Increased levels of all peroxiredoxins except Prdx4 were shown in Huh7.5 cells infected with hepatitis C virus that also belongs to this family [144]. Noteworthy that different kinetics of expression was noted for different isoforms of the enzyme. Upregulation of Prdx1 occurs in the liver of chronic hepatitis B patients, indicating that changes in its expression are not restricted to flaviviruses [145]. Another member of the *Flaviviridae* family—the Zika virus was reported not to affect the expression of Prdx2, whereas the status of other peroxiredoxins, unfortunately, was not accesses [155].

Persistent lymphocytic choriomeningitis virus (PLCV) is a neglected pathogen that causes severe prenatal infection. It belongs to mammarenoviruses, as the abovementioned Junine mammarenovirus. PLCV was shown to downregulate the expression of several peroxiredoxins, including 2, 4, and 6 [146]. A decrease in Prdx1 expression was also observed in cells infected with the rabies virus [147].

A different mechanism for the regulation of peroxiredoxin expression was observed for the human immunodeficiency virus, the protease of which recognizes Prdx2 as its substrate [156]. However, this is not accompanied by a decrease in its expression in the plasma of the patients. Similar degradation of Prdx6 occurred in the case of porcine picornavirus 3C protease [128]. As mentioned above, the latter is required to evade its antiviral activity.

### 7.3. Peroxiredoxins as Biomarkers

There are several reports on the association of changes in Prdx levels with some development of certain pathologies. In these cases, peroxiredoxins can be evaluated as biomarkers of viral infections or virus-mediated diseases. In particular, for patients with HIV—associated dementia (HAD), a decrease in Prdx3 levels in monocytes was shown, which could contribute to the development of neuronal damage through the loss of hydrogen peroxide scavenging capabilities [157]. Decreased levels of Prdx2 were seen in the cerebral spinal fluid of such AIDS patients [158]. Moreover, the presence of cross-reactive antibodies to p24-(gag) protein of another lentivirus, human T-lymphotropic virus type-1 (HTLV-1), and peroxiredoxin 1 was noted [159]. It can be speculated that scavenging of this antioxidant enzyme can also contribute to the development of virus-associated neurological disorders. In some patients with HCV-associated liver cancer, autoantibodies to Prdx6 were also detected [160].

Enhanced expression of Prdx3 in livers of chronic hepatitis B patients with advanced fibrosis was also reported [161]. An independent study suggested that increased levels of Prdx2 in plasma can be used to detect early-stage fibrosis in patients with hepatitis B virus infection [162]. Peroxiredoxins are also associated with the development of liver cancer during viral hepatitis infection. One study reported decreased expression of Prdx2 in tumors and an increase in peritumor areas of livers in HBV-associated hepatocellular carcinoma patients [163]. This can indicate the role of this enzyme as a tumor suppressor. However, the opposite situation occurs in liver cancer patients with chronic hepatitis C, albeit for different isoforms of the enzyme [164]. Interestingly, women with hepatocellular carcinoma (HCC) exhibit elevated expression of Prdx3, whereas men—of Prdx1. Low levels of Prdx3 can be regarded as a negative prognostic factor for patients with HBV or HCV-related hepatocellular carcinoma [165]. In patients with chronic hepatitis C without liver cancer, decreased levels of Prdx1 and Prdx5 in plasma were reported [166].

Prdx1 can be used as a negative prognostic marker in patients with Dengue virus, i.e., of the development of hemorrhagic fever [150]. Five-fold higher levels of this enzyme were found PBMC of patients with dengue hemorrhagic fever compared to patients with dengue fever.

Prdx 1 and Prdx2 are also released from the cells under lipopolysaccharide (LPS) treatment and influenza virus infection, adding them to a list of biomarkers of inflammation [144].

Another piece of evidence was given by a very strong association of the rs7082598 single nucleotide polymorphism (SNP) in a gene encoding Prdx3 with cancer incidence in patients with human papilloma virus (HPV) [167]. Thus, this SNP can be used in the future to stratify the patients for more close monitoring for this type of cancer. However, it should be noted that high levels of Prdx3 expression in cervical cancer cells are not induced by the virus [168].

## 8. Conclusions and Future Perspectives

In the current review, we have briefly summarized the properties of all human peroxiredoxins, and thus, showed their significance in scavenging hydrogen and organic peroxides. In addition, a current understanding of the regulatory functions of these antioxidant enzymes has been demonstrated. These data demonstrate that peroxiredoxins are the proteins that can transfer signals from reactive oxygen species to specific proteins. However, the status of peroxiredoxins during various viral infections and their roles in the virus life cycle and development of virus-associated pathologies remains mostly obscure.

To date, various viruses have been shown to trigger ROS production. However, almost all studies are based on a classical concept of oxidative stress—an imbalance between total ROS production and the antioxidant capacity of a cell. It hampers search for origins of ROS overproduction and does not identify cellular proteins that are controlled by specific cysteine oxidation—the subjects of modern redox biology. Since peroxiredoxins have the highest potential for peroxide neutralization, analysis of their oxidation in cells and their compartments in the future may reveal the exact sites for ROS production that are affected by infections. A single such study revealed oxidation of peroxiredoxins 1, 3, and 4 during RSV infection, suggesting the generation of ROS in their proximity [140]. In addition, it may identify a list of Prdx partner proteins that may be (in)activated through redox relay mechanisms via forming intermolecular cysteine bonds. Moreover, the absence of data on peroxiredoxin expression and oxidation status during viral infections does not make conclusions about the status of antioxidant defense systems of a host cell/tissue. Finally, a unique ability of several peroxiredoxins to form oligomers upon overoxidation or other posttranslational modifications with concomitant export from the infected cells may identify novel biomarkers for the virus-associated pathologies. Clearly, all these questions merit further studies in the coming years. 

## Figures and Tables

**Table 1:** Intracellular localization of peroxiredoxins.

Enzyme	Intracellular Localization
Prdx1	Cytoplasm, nucleus
Prdx2	Cytoplasm, nucleus, plasma membrane
Prdx3	Mitochondria
Prdx4	Endoplasmic reticulum
Prdx5	Mitochondria, peroxisomes, cytoplasm
Prdx6	Acidic organelles, mitochondria, nucleus

**Table 2:** Transcription factors that control the expression of peroxiredoxins.

Enzyme	Transcription Factor Regulating Prdx Expression
Prdx1	Nrf2, AP1, NF-κB
Prdx2	FoxO3a, NF-κB
Prdx3	Nrf2, FoxO3a, Nrf1/GABP, cMyc
Prdx4	Unknown
Prdx5	Nrf2, FoxO3a, Nrf1/GABP, GATA1
Prdx6	Nrf2, Sp1, C/EBPβ

**Table 4:** Expression of peroxiredoxins during viral infections.

Virus	Peroxiredoxin	Model	Ref
HIV	Prdx4 ↓	T-cells from patients	[129]
Porcine picornavirus, foot and mouth disease virus	Prdx6 ↓	MEFs, immortalized and transformed cells (various MOI)	[128]
Human papilloma virus	Prdx1,2 ↑	Overexpression of virus oncoproteins	[135,141]
Epstein–Barr virus	Prdx1 ↑	Overexpression of EBNA1 protein	[142]
Dengue virus	Prdx1 ↑	PBMC from patients	[143]
Hepatitis C virus	Prdx1,2,3,5,6 ↑	Hepatoma Huh7.5 cells	[144]
Hepatitis B virus	Prdx1 ↑	Liver of transgenic mice	[145]
Persistent lymphocytic choriomenegitis virus	Prdx2,4,6 ↓	Immortalized and tumor cell lines	[146]
Rabies virus	Prdx1 ↓	Lymphocytes from mice	[147]

PBMC: peripheral blood mononuclear cells; MEF: mouse embryonic fibroblasts. ↓: downregulation; ↑: upregulation.
